# Cellular Levels of HIV Unspliced RNA from Patients on Combination Antiretroviral Therapy with Undetectable Plasma Viremia Predict the Therapy Outcome

**DOI:** 10.1371/journal.pone.0008490

**Published:** 2009-12-31

**Authors:** Alexander O. Pasternak, Suzanne Jurriaans, Margreet Bakker, Jan M. Prins, Ben Berkhout, Vladimir V. Lukashov

**Affiliations:** 1 Department of Medical Microbiology, Laboratory of Experimental Virology, Center for Infection and Immunity Amsterdam (CINIMA), Academic Medical Center of the University of Amsterdam, Amsterdam, The Netherlands; 2 Department of Medical Microbiology, Laboratory of Clinical Virology, Academic Medical Center of the University of Amsterdam, Amsterdam, The Netherlands; 3 Department of Internal Medicine, Division of Infectious Diseases, Tropical Medicine and AIDS, Academic Medical Center of the University of Amsterdam, Amsterdam, The Netherlands; George Washington University, United States of America

## Abstract

**Background:**

Combination antiretroviral therapy (cART), the standard of care for HIV-1 infection, is considered to be successful when plasma viremia remains below the detection limit of commercial assays. Yet, cART fails in a substantial proportion of patients after the apparent success. No laboratory markers are known that are predictive of cART outcome in initial responders during the period of undetectable plasma viremia.

**Methodology/Principal Findings:**

Here, we report the results of a retrospective longitudinal study of twenty-six HIV-infected individuals who initially responded to cART by having plasma viremia suppressed to <50 copies/ml. Eleven of these patients remained virologically suppressed, whereas fifteen experienced subsequent cART failure. Using sensitive methods based on seminested real-time PCR, we measured the levels of HIV-1 proviral (pr) DNA, unspliced (us) RNA, and multiply spliced RNA in the peripheral blood mononuclear cells (PBMC) of these patients at multiple time points during the period of undetectable plasma viremia on cART. Median under-therapy level of usRNA was significantly higher (0.43 log_10_ difference, *P* = 0.0015) in patients who experienced subsequent cART failure than in successfully treated patients. In multivariate analysis, adjusted for baseline CD4^+^ counts, prior ART experience, and particular cART regimens, the maximal usRNA level under therapy was the best independent predictor of subsequent therapy failure (adjusted odds ratio [95% CI], 24.4 [1.5–389.5], *P* = 0.024). The only other factor significantly associated with cART failure was prior ART experience (adjusted odds ratio [95% CI], 12.3 [1.1–138.4], *P* = 0.042). Levels of usRNA under cART inversely correlated with baseline CD4^+^ counts (*P* = 0.0003), but did not correlate with either baseline usRNA levels or levels of prDNA under therapy.

**Conclusion:**

Our data demonstrate that the level of HIV-1 usRNA in PBMC, measured in cART-treated patients with undetectable plasma viremia, is a strong predictive marker for the outcome of therapy.

## Introduction

Combination antiretroviral therapy (cART), the standard of care for human immunodeficiency virus type 1 (HIV-1) infection [1]–[3], is an exemplary success of modern medicine, as it has resulted in a dramatic decrease in HIV-related morbidity and mortality [4], [5]. Since the introduction of cART, the response to therapy has dramatically improved, with currently most patients reaching HIV RNA levels in plasma that are below the limit of detection of modern commercial assays (<50 copies/ml). Despite these encouraging results, failure of ART is still a common problem. In the Netherlands, the annual proportion of previously therapy-naïve patients who experience failure increased from 6% to 9% in the period between 1997 and 2007 (F. de Wolf. The HIV epidemic in the Netherlands. The 2nd Netherlands Conference on HIV Pathogenesis, Prevention and Treatment, 25 November 2008, Amsterdam, The Netherlands).

In initial cART responders, previous ART experience and specific drug regimens are associated with subsequent therapy failure (virological rebound, VR) [6]–[11]. In addition, several baseline parameters (plasma HIV-1 viremia, CD4^+^ counts, HIV-1 DNA level in PBMC) were reported to be associated with the risk of VR [6], [12], [13]. However, no laboratory markers have yet been identified that are predictive of cART outcome in initial responders during the period of undetectable plasma viremia.

Whether HIV-1 continues to replicate in patients on cART is a matter of considerable debate [14]–[26]. The best evidence for residual HIV-1 replication comes from the studies on viral evolution [17], [18], [20] [22], which demonstrated patient-specific differences in HIV-1 evolution under cART. If residual viral replication continues in some patients on cART but not in others, the former are more prone to develop drug-resistance mutations and, as a consequence, fail therapy. Hence, quantifying the levels of intracellular HIV-1 RNA and DNA in patients on cART could provide hints as to why some patients fail therapy.

We have recently developed sensitive seminested real-time PCR methods based on TaqMan chemistry for quantitation of intracellular HIV-1 proviral (pr) DNA and both unspliced (us) and multiply spliced (ms) RNA forms [27]. Using these methods, we have recently demonstrated a singnificant longitudinal increase in levels of usRNA in PBMC of the majority of untreated patients with steady-state plasma RNA loads in the asymptomatic phase of HIV-1 infection (Pasternak *et al*., manuscript in preparation). This finding indicates that in untreated patients, HIV-1 usRNA load in PBMC may be a more sensitive prognostic indicator than viral RNA load in plasma. Here, we used these methods to study the predictive value of intracellular levels of viral RNA and DNA for the virological outcome of therapy.

## Results

Cellular HIV-1 load (prDNA, usRNA, and msRNA) was quantified in PBMC at baseline and at multiple time points during the period of undetectable HIV-1 plasma viremia on cART (“the eclipse phase”) in patients who were successfully treated with cART (“successes”) and those who experienced VR on cART after achieving undetectable HIV-1 load in plasma (“failures”). UsRNA and prDNA were detected and quantified in all baseline PBMC samples and in 94% of the under-therapy PBMC samples. MsRNA could be detected and quantified in 83% of the baseline samples, but only in 16% of the under-therapy samples (Table S1), precluding any quantitative comparison of msRNA levels under cART between the patient groups. To account for the possible effects of mismatches in the primer and probe binding regions on the efficiency of real-time PCR, all quantified amounts of prDNA and usRNA in the baseline and under-therapy PBMC samples were normalized to the individual mismatch-related quantification errors (see Methods and Figure S1).

None of the baseline characteristics, except for prior ART experience (*P* = 0.015) and baseline CD4^+^ count (*P* = 0.019), was significantly associated with subsequent VR (Table 1). No significant correlations were observed in any pair of the baseline quantitative parameters, except for usRNA and msRNA (*P* = 0.023, *r_s_* = 0.51). Among the antiretroviral drugs received by the patients, only the use of nelfinavir was significantly associated with VR (*P* = 0.024), confirming earlier reports [9], [11]. No significant association of the presence of drug-resistant HIV-1 strains at baseline and subsequent VR was apparent.

**Table 1:** Baseline characteristics of patients and cART regimens.

			All patients	Future failures	Successes	*P* a
**Baseline parameters**	Date of cART initiation		06.1997 (12.1996–02.1998)^b^	10.1997 (01.1997–02.1998)	05.1997 (01.1997–12.1997)	0.82
	Age, years		41 (32–47)	45 (36–47)	34 (32–45)	0.23
	Plasma RNA, log_10_ copies/ml		4.48 (4.03–4.82)	4.59 (3.94–4.94)	4.48 (4.21–4.52)	0.64
	UsRNA, log_10_ copies/µg total RNA		4.52 (4.21–4.99)	4.53 (4.13–5.01)	4.50 (4.33–4.91)	0.73
	MsRNA, log_10_ copies/µg total RNA		3.04 (2.83–3.24)	3.08 (2.83–3.21)	3.01 (2.84–3.30)	1
	PrDNA, log_10_ copies/10^6^ PBMC		3.86 (3.46–4.15)	3.91 (3.64–4.20)	3.75 (3.37–4.08)	0.56
	CD4^+^ T cell count, cells/mm^3^ blood		270 (163–365)	250 (140–280)	370 (230–470)	0.019
**Prior ART experience**	Naive		13 (50%)^c^	4 (27%)	9 (82%)	0.015
	Experienced		13 (50%)	11 (73%)	2 (18%)	
		Dual NRTI	8 (62%)	7 (64%)	1 (50%)	
		Triple NRTI	1 (8%)	1 (9%)	0	
		cART	4 (31%)	3 (27%)	1 (50%)	
**Baseline drug resistance**	General	NRTI	10 (43%)	7 (54%)	3 (30%)	0.40
		PI	4 (20%)	1 (10%)	3 (30%)	0.58
	To the cART in this study	NRTI	7 (30%)	4 (31%)	3 (30%)	1
		PI	2 (10%)	1 (10%)	1 (10%)	1
**cART regimen**	NRTI 1	Didanosine	8 (31%)	7 (47%)	1 (9%)	0.084
		Lamivudine	18 (69%)	8 (53%)	10 (91%)	
	NRTI 2	Stavudine	19 (73%)	11 (73%)	8 (73%)	1
		Zidovudine	7 (27%)	4 (27%)	3 (27%)	
	PI/NNRTI	Nelfinavir	6 (23%)	6 (40%)	0	0.024^d^
		Nevirapine	7 (27%)	4 (27%)	3 (27%)	1
		Indinavir	4 (15%)	3 (20%)	1 (9%)	0.61
		Ritonavir	2 (8%)	0	2 (18%)	0.17
		Ritonavir + Nevirapine	1 (4%)	1 (7%)	0	1
		Ritonavir + Saquinavir	4 (15%)	1 (7%)	3 (27%)	0.28
		Ritonavir + Indinavir	2 (8%)	0	2 (18%)	0.17

a Failures and successes were compared.

b Median values and interquartile ranges are shown for the continuous variables.

c Numbers and percentages of patients are shown for the discrete variables.

d The use of any individual PI/NNRTI was compared between the patient groups against the use of all the other PI/NNRTI combined.

**Figure 1:**
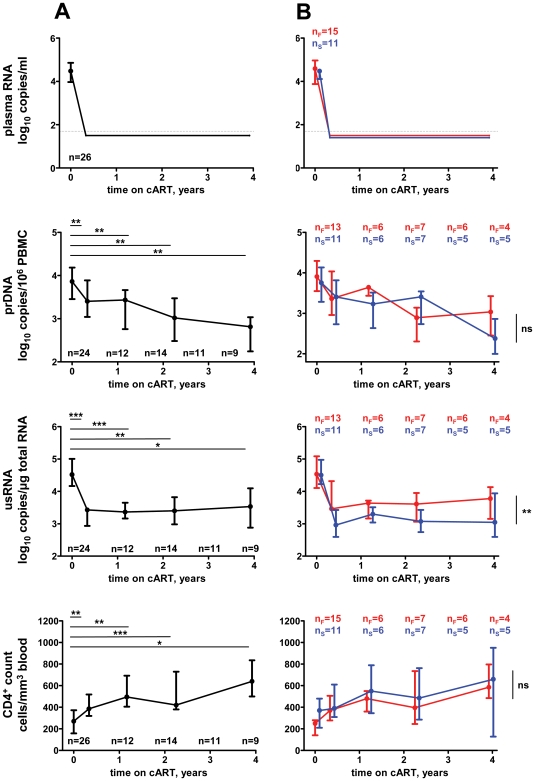
Levels of virological parameters and CD4^+^ counts. Time points on the X axes correspond to the median times from cART initiation of PBMC samples for every of the sampling time groups (see Methods). Numbers of patients in every sampling time group are indicated on the graphs. For the upper panels, total numbers of patients are indicated. Median values and interquartile ranges of the studied parameters are indicated. Thin dotted lines on the upper panels indicate the limit of detection of the modern commercial assays (50 copies/ml). Levels of statistical significance are depicted on the graphs and signify the following *P* values: ***, *P*<0.001; **, 0.001<*P*<0.01; *, 0.01<*P*<0.05; ns (not significant), *P*>0.05. (A) All patients combined. Parameters were compared between baseline and time on cART-grouped PBMC samples. (B) Patient groups (red, failures; blue, successes). Parameters were compared between failures and successes. Numbers of failures and successes are indicated by n_F_ and n_S_, respectively. To prevent overlap, data sets are nudged between the patient groups.

Figure 1 shows levels of HIV-1 prDNA, usRNA, and CD4^+^ counts at baseline and at four time points during the eclipse phase in all patients (Figure 1A), and the failures and successes separately (Figure 1B). Significant changes from baseline in levels of all parameters were observed after cART initiation (Figure 1A). The median (IQR) drop in prDNA level between the baseline and the first time point on cART was 0.49 (0.40–0.78) log_10_ (*P* = 0.002), whereas the corresponding drop in usRNA level was 1.05 (0.90–1.50) log_10_ (*P*<0.001). The difference between the drops in prDNA and usRNA levels was significant (*P* = 0.002). Subsequently, we used repeated measures analysis of variance (RM-ANOVA) to compare the under-therapy levels of HIV-1 prDNA, usRNA, and CD4^+^ count between the patient groups. Baseline values were excluded from this analysis. No significant differences were observed between the failures and successes in the under-therapy level of prDNA and CD4^+^ count (figure 1B). Conversely, there was a statistically significant difference in the level of usRNA under cART between the patient groups (*P* = 0.0015), the median usRNA level of failures being 0.43 log_10_ higher than that of successes. This difference was established early after the start of cART and observed throughout the eclipse phase (Figure 1B).

**Figure 2:**
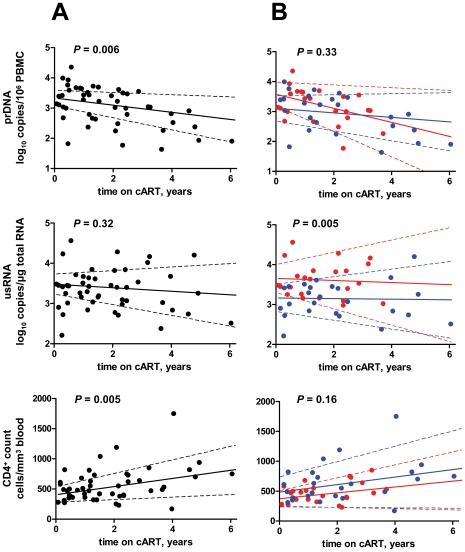
Longitudinal trends of the virological parameters and CD4^+^ count during the eclipse phase. The dots represent all the PBMC samples used in this study. Best-fit lines and their 95% CI are shown by solid and dashed lines, respectively. (A) All patients combined. Levels of statistical significance of the longitudinal trends are shown on the graphs. (B) Patient groups (red, failures; blue, successes). Levels of statistical significance of the comparison of the parameters between the patient groups are shown on the graphs.

Next, we estimated average longitudinal trends of the parameters during the eclipse phase by fitting linear mixed models (Figure 2). Again, baseline values were excluded from this analysis. Trends observed for prDNA and CD4^+^ count were statistically significant (*P* = 0.006 and *P* = 0.005, respectively), with prDNA declining on average by 0.12 log_10_/year, and CD4^+^ T cell number increasing by 68 cells/mm^3^ blood/year. Levels of usRNA did not significantly change over time in all patients (Figure 2A), and the separate patient groups (Figure 2B). In this analysis, statistically significant difference in the levels of virological parameters between failures and successes was again observed only for the levels of usRNA (*P* = 0.005; Figure 2B).

**Figure 3:**
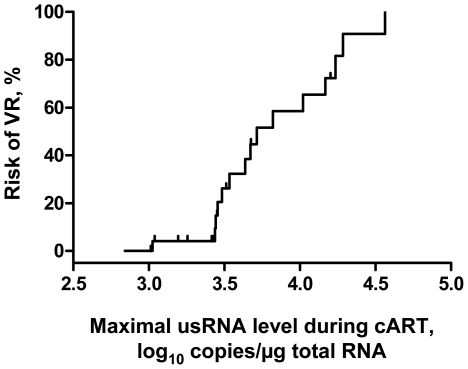
The risk of VR according to the maximal usRNA value during the eclipse phase. Kaplan-Meier plot shows the risk of VR as a function of maximum usRNA level measured during the eclipse phase. Small vertical bars show successfully treated patients.

Based on the observation that higher levels of usRNA are associated with higher risk of VR, we used the maximal value of usRNA measured during the eclipse phase to determine a clinically relevant cutoff value of HIV-1 usRNA in PBMC for the risk of VR. Area under the receiver operating characteristic (ROC) curve was 0.800 (95% CI, 0.598–0.929; *P* = 0.0006), indicating the ability of the maximal level of usRNA under therapy to correctly predict VR in 80% of random cases, and the cutoff value of usRNA was 3.43 log_10_ copies/µg total RNA (∼2700 copies/µg total RNA). As shown by the Kaplan-Meier plot (Figure 3), whereas the risk of VR remained low (<5%) for maximal under-therapy values of usRNA smaller than the cutoff value, the risk increased rapidly for higher values.

Next, we stratified the patients into two groups based on the determined cutoff value of usRNA. Of the eight patients whose usRNA level under cART did not reach the cutoff value, only one experienced VR, whereas VR was observed in 14 out of 18 patients whose maximal usRNA level was higher than the cutoff value (unadjusted odds ratio (OR), 24.5; 95% CI, 2.3–262.7; *P* = 0.0033). Subsequently, maximal level of usRNA under cART, along with other factors associated with the VR in univariate analyses with significance levels <0.1 (baseline CD4^+^ count, prior ART experience, use of nelfinavir as PI, use of didanosine as NRTI), were included in the multivariate logistic regression analysis. Maximal level of usRNA under cART was independently predictive of the therapy outcome (adjusted OR for VR (95% CI), 24.4 (1.5–389.5), *P* = 0.024). The only other factor significantly associated with VR was prior ART experience (adjusted OR (95% CI), 12.3 (1.1–138.4), *P* = 0.042). Area under curve of the logistic regression was 0.882 (95% CI, 0.695–0.974, *P* = 0.0004), meaning that the predictive power of the multivariate model was 88.2%.

**Figure 4:**
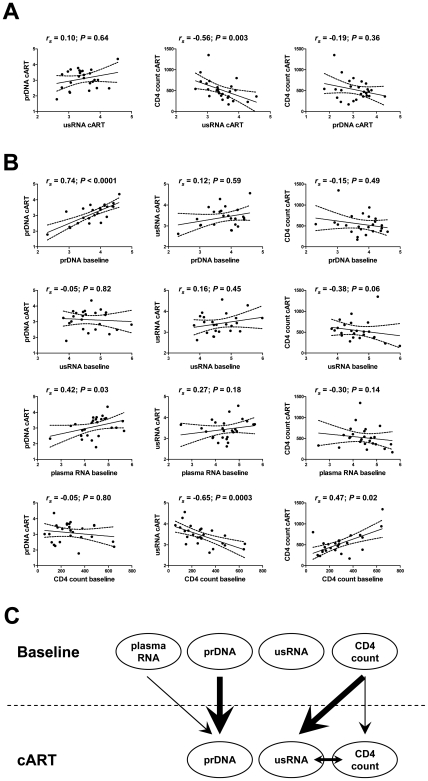
Correlations of virological parameters and CD4^+^ count. Best-fit lines and 95% CI are shown by solid and dashed lines, respectively. Spearman correlation coefficients (*r_s_*) and corresponding *P* values are indicated within the graphs. Mean values of the quantified under-therapy parameters per patient (geometric means of log_10_-transformed values of usRNA and prDNA load and arithmetic means of CD4^+^ count) were used. The units of measurement are log_10_ copies/10^6^ PBMC for prDNA, log_10_ copies/µg total RNA for usRNA, log_10_ copies/ml for plasma RNA, and cells/mm^3^ blood for CD4^+^ count. (A) Correlations between the under-therapy levels of the studied parameters. (B) Correlations between the baseline and under-therapy levels. (C) Schematic representation of the correlations shown in panels A and B. Arrows indicate significant correlations, and thickness of arrows indicates levels of statistical significance: thick arrows, *P*<0.001; intermediate arrows, 0.001<*P*<0.01; thin arrows, 0.01<*P*<0.05.

Finally, correlation tests were performed between pairs of the under-therapy levels of the studied parameters (Figure 4A) and combinations of the baseline and under-therapy levels (Figure 4B). Figure 4C summarizes the observed correlations. No significant correlation was observed between the under-therapy levels of prDNA and usRNA, but levels of usRNA under cART inversely correlated both with CD4^+^ count under therapy (*r_s_* = −0.56, *P* = 0.003) and, even more strongly, with baseline CD4^+^ count (*r_s_* = −0.65, *P* = 0.0003). Of note, no significant correlation was observed between the baseline and under-therapy levels of usRNA, whereas baseline and under-therapy levels of prDNA strongly correlated between each other (*r_s_* = 0.74, *P*<0.0001). As subsequently assessed by Pearson analysis, 40% of the variance of usRNA level under cART could be explained by the baseline CD4^+^ count, but only 6% of its variance could be explained by the baseline usRNA level. By contrast, 59% of the variance of prDNA level under cART could be explained by the baseline prDNA level.

Due to the low detectability of msRNA in the under-therapy PBMC samples, we could not assess correlations of this marker with the other parameters. However, median level of usRNA in the msRNA^+^ under-therapy PBMC samples was 0.48 log_10_ higher than usRNA level in the msRNA^−^ samples (*P* = 0.034, Mann-Whitney test), suggesting that the correlation between levels of usRNA and msRNA, observed at baseline, exists also under cART. The levels of msRNA in PBMC before the start of cART were, on average, 1.5 log_10_ lower than those of usRNA (Table 1). Interestingly, exactly the same msRNA/usRNA ratio was observed in the msRNA^+^ under-therapy PBMC samples (Table S1). If this ratio holds for the samples where msRNA was undetectable, the median level of msRNA in the under-therapy PBMC samples should theoretically be ∼1.9 log_10_ copies/µg total RNA, which is below the average limit of detection of our msRNA assay, explaining the low (16%) detectability of msRNA.

## Discussion

The main clinical objective of cART is suppression of HIV-1 plasma viremia to below the lowest existing detection limit of commercial assays. In most patients on cART, this objective is achieved, and therefore plasma viremia in these patients cannot be informative about the subsequent outcome of therapy. Hence, additional markers have to be identified that are associated with therapy outcome in patients with fully suppressed plasma viremia. In this study, we demonstrated that the level of HIV-1 usRNA in PBMC from such patients is predictive of subsequent VR. To the best of our knowledge, this is the first report showing that a viral parameter–measured in a patient on cART during the period of undetectable plasma viremia–is predictive of the outcome of therapy. Several reports have indicated the presence of HIV-1 mRNA in PBMC from patients on cART [28]–[34]. However, the mere presence of intracellular viral RNA does not necessarily imply residual virus replication, and may instead reflect production of virus from stable reservoirs without new replication cycles. In contrast, our observation that patients with higher levels of usRNA in PBMC were more prone to failing cART might link higher cellular HIV-1 RNA load in these patients with virus replication under therapy, which, in turn, resulted in selection of drug-resistance mutations. Acquired drug-resistance mutations were indeed observed in all plasma samples sequenced after failure (data not shown). It remains to be investigated whether drug-resistance mutations can indeed be detected in PBMC of these patients during the eclipse phase. Low-level plasma viremia was recently detected by ultrasensitive assays in most patients on cART [15], [24]–[26]. Whether this residual viremia reflects ongoing virus replication despite therapy, or the production of virus from stable reservoirs without new replication cycles, is controversial [15], [23], [26], [35], [36]. The possible association of the residual plasma viremia with the outcome of cART and its correlation with the levels of usRNA in PBMC remain to be studied.

Latently infected resting CD4^+^ T cells are considered to be an extremely stable viral reservoir in patients on suppressive cART, and a major barrier to HIV-1 eradication [16], [21], [37]–[39]. Occasionally, these cells may become reactivated, as a response to antigens or cytokine induction [21]. Because unfractionated PBMC were used in our study, we could not discern whether the HIV-1 usRNA detected in PBMC was derived from resting or activated CD4^+^ T cells, but we assume that the majority of usRNA was derived from activated CD4^+^ T cells, productively infected with HIV-1. This is supported by the observed differences in longitudinal trends between prDNA and usRNA after the start of cART. Whereas levels of prDNA gradually diminished throughout the whole follow-up period, those of usRNA first sharply declined after the start of therapy and reached the plateau during the eclipse phase (Figure 1). These differences can be explained by the fact that prDNA levels reflect the size of the proviral archive, whereas levels of usRNA reflect the amounts of productively infected cells at every moment in time. Similar differences in decay kinetics between HIV-1 DNA and RNA after the start of ART have been reported by others [29], [32], [40], [41] and are consistent with the observations that latently infected cells harboring inactive HIV proviruses are cleared after the start of therapy much slower than productively infected cells [19], [39], [42]. During the eclipse phase, after the initial drop in the amount of productively infected cells, very few cells among the total bulk of latently infected cells would become reactivated at any given moment. However, because activated CD4^+^ T cells can contain up to 4000 copies of HIV-1 usRNA/cell [43], this small fraction of cells may be largely “responsible” for the usRNA levels detected in this study. Then, the observed difference in the usRNA levels between failures and successes may be shaped by the difference in the relative amounts of latently infected resting CD4^+^ T cells that become reactivated to the productively infected phenotype and escape immunological clearance. Importantly, we have observed that usRNA levels under cART strongly inversely correlated with baseline CD4^+^ counts but did not correlate with either HIV-1 prDNA levels under therapy or baseline levels of HIV-1 usRNA, prDNA, or plasma RNA (Figure 4). Conversely, a strong correlation has been observed between baseline and under-therapy levels of HIV-1 prDNA. Thus, the relative amount of cells expressing viral RNA at any moment during cART (reflected in the usRNA level) might be defined, to a large extent, by the immunological state of the patient before therapy (reflected in the CD4^+^ count), and not simply by the size of viral reservoir (reflected in the levels of prDNA). Consequently, level of HIV-1 usRNA in PBMC under cART, which we found to be (i) strongly inversely associated with baseline CD4^+^ count, and (ii) strongly predictive of subsequent VR, provides a missing link explaining the previously observed inverse association between the baseline CD4^+^ count and the risk of therapy failure [6], [12]. Remarkably, the level of usRNA under cART was a better predictor of therapy failure than the CD4^+^ count at baseline. Although baseline CD4^+^ count could explain 40% of the variance of usRNA level under cART, other parameters known to be associated with VR, such as adherence to cART, may also influence the levels of usRNA.

There are certain limitations to this study. First, our usRNA assay does not distinguish between genuine intracellular HIV-1 usRNA and cell-associated virion RNA. However, Fischer *et al.* [28] have shown that in patients on cART with plasma viremia suppressed to <50 copies/ml, extracellular fraction comprised, on average, 0.6% of total quantified usRNA, which is negligible. Secondly, this study included patients who started cART, on average, in 1997, and their therapy regimens would currently be considered suboptimal as a first-line therapy in the developed world, mainly due to toxicity issues [44]. While generalization of our findings requires replication of the results in patients treated with current antiretroviral drugs, we would like to stress that: (i) antiretroviral drugs received by patients in this study are still widely used in the resource-limited settings [44], and a substantial proportion of them is recommended for use as a first-line therapy in the developing world by the World Health Organization [45], and (ii) cART regimens used in this study suppressed plasma viremia in all of our patients to <50 copies/ml, a clinically accepted detection limit of the modern ultrasensitive assays for HIV-1 RNA detection. Furthermore, in PBMC of patients treated with cART in 2006–2007, levels of usRNA were essentially similar to those observed in this study (A.O.P. and V.V.L., unpublished data).

In summary, our findings demonstrate that the level of HIV-1 unspliced RNA in PBMC is a strong predictive marker for the outcome of therapy in cART-treated patients. Use of this quantitative assay in the standard laboratory practice could aid in monitoring the course of cART and facilitate the early detection of drug-resistant escape mutants before the actual failure of the therapy.

## Methods

### Patients and Patient Samples

We have used archival PBMC samples from HIV-1 infected individuals who were participating in the Amsterdam Cohort Studies (ACS) on HIV infection and AIDS. The ACS have been conducted in accordance with the ethical principles set out in the Declaration of Helsinki, and written informed content has been obtained prior to sample collection. The study has been approved by the ACS committee. The ACS have been approved by the Medical Ethical Committee of the Academic Medical Center.

We have selected 26 HIV-1 infected individuals who received cART between 1996 and 2002, and have initially responded to cART by showing undetectable HIV-1 RNA loads in plasma (<50 copies/ml). cART was defined as at least a triple-therapy regimen, consisting of two nucleoside reverse transcriptase inhibitors (NRTI) and at least one protease inhibitor (PI) or non-nucleoside reverse transcriptase inhibitor (NNRTI). All patients were men infected by HIV-1 subtype B strains. Two groups of patients were selected, matched by median calendar year of cART initiation: successfully treated patients (n = 11) and patients who experienced VR after initially responding to therapy (failures on cART; n = 15). Individuals treated with cART, in whom HIV-1 plasma viremia dropped to the undetectable levels (<50 copies/ml) and remained undetectable for the whole period of the therapy (with the minimum of one year), were considered to have virological success. Occasional “blips” (transient episodes of detectable plasma viremia) of <500 copies/ml, preceded and followed by measurements of <50 copies/ml, were allowed. Individuals treated with cART for at least six months, in whom HIV-1 plasma viremia has dropped to the undetectable levels after the start of therapy, has been undetectable for at least three months after that, and subsequently became detectable (>50 copies/ml in two consecutive measurements or >500 copies/ml in any measurement), were considered to experience VR (cART failure). No difference was observed in the frequency of blips between the patient groups.

Baseline was defined as the date of start of cART. The follow-up period was calculated from the start of therapy until the date of first measurement of plasma viremia above the detection limit (for failures), or until the date of last measurement of plasma viremia under the current therapy regimen (for successes). Median (IQR; range) follow-up periods were 2.30 (1.58–3.48; 0.62–6.04) years for all patients, 2.16 (1.40–2.85; 0.62–3.97) years for failures, and 2.48 (2.11–4.68; 1.32–6.04) years for successes. During the follow-up period, plasma viremia was monitored at least every four months, and cellular HIV-1 load was quantified at multiple time points (Table S1). The numbers of under-therapy PBMC samples were 24 for failures and 25 for successes. There were no significant differences between the patient groups in the PBMC sampling times from the start of therapy (*P* = 0.35). For every PBMC sample used in this study, concurrent measurements of plasma viremia and CD4^+^ count were available.

### Quantitation of HIV-1 Load in PBMC and Plasma Samples

For quantitation of cellular HIV-1 RNA and DNA load, PBMC were isolated by standard Ficoll-Hypaque density gradient centrifugation and frozen in aliquots in liquid nitrogen. Total cellular nucleic acids were extracted from PBMC samples (∼10^6^ PBMC was used for one extraction) according to the isolation method of Boom *et al*. [46], eluted in water, and frozen in aliquots at −80°C until further processing. HIV-1 prDNA and both forms of cellular HIV-1 RNA (usRNA and msRNA) were quantified by seminested real-time PCR, as described earlier [27]. The eluted cellular DNA was directly subjected to two rounds of PCR amplification: a limited-cycle pre-amplification step and a real-time PCR step, using seminested primers. For RNA quantitation, the eluted RNA samples were first subjected to DNase treatment (DNA-free kit, Ambion), to remove HIV-1 prDNA which could interfere with the quantitation, and subsequently to reverse transcription (RT). For both usRNA and msRNA assays, two rounds of amplification with seminested primers were performed on the resultant cDNA. For all assays, no positive signals have been obtained from the negative controls, as well as from the –RT controls for RNA assays, which were included in the quantitation. The amounts of PBMC-derived HIV-1 DNA and RNA were normalized to total cellular inputs, which were quantified in separate real-time PCR by using the detection kits for either beta-actin or ribosomal RNA, respectively (both–Applied Biosystems), and expressed either as number of copies per 10^6^ PBMC for prDNA, or as number of copies per µg total RNA for usRNA and msRNA. The sensitivity of all three assays was four copies per reaction, which translated into approximately 40 copies/10^6^ PBMC for the prDNA assay and 100 copies/µg total RNA for RNA assays (actual detection limits depended on the total cellular inputs of the PBMC samples), and the linear range was at least five orders of magnitude. The reproducibility and accuracy of these assays have been demonstrated earlier [27]. For further validation of the assays, prDNA and usRNA were quantified in 28 PBMC samples, randomly selected from the pool of samples from patients under cART, in triplicates (including independent extraction and RT-PCR). The mean coefficients of variation, calculated on log_10_-transformed values, were 5.31% (95% CI, 4.41–6.31%) for the prDNA assay, and 2.29% (95% CI, 1.35–5.31%) for the usRNA assay.

Plasma viremia was quantified using commercial ultrasensitive assays with limits of detection of 40 or 50 copies/ml (Quantiplex HIV-1 RNA 3.0, Bayer Diagnostics, or M2000RT, Abbott Molecular), according to manufacturers' recommendations. For those patients who were treated with cART before the year 2000 (when ultrasensitive assays for plasma HIV-1 RNA quantitation were introduced in our laboratory), HIV plasma viremia was remeasured using the modern ultrasensitive assays.

### Determination of Baseline Drug Resistance

To determine the presence of drug-resistant HIV-1 strains at baseline, we used prDNA extracted from the baseline PBMC samples as described above. Regions corresponding to the *pol* gene of HIV-1 were amplified in the nested PCR with the following primers: 5′Prot I (5′-AGGCTAATTTTTTAGGGAAGATCTGGCCTTCC-3′) and 3′ET-21 (5′-AGCTGGCTACTATTTCTTTTGCTACTACAGGTGG-3′) (pol-PCR I), and 5′Prot II (5′-TCAGAGCAGACCAGAGCCAACAG-3′) and 3′RT20 (5′- CTGCCAGTTCTAGCTCTGCTTC-3′) (pol-PCR II). PCR products were sequenced directly using the primers of the pol-PCR II. The sequenced fragments contained all 99 codons of protease and the first 284 codons of reverse transcriptase. Analysis of drug-resistance mutations was carried out using the Geno2pheno software (Max-Planck-Institut for Informatics, Saarbrücken, Germany, available online at http://www.geno2pheno.org/), which includes the algorithm for the determination of resistance for every antiretroviral drug individually by using the Z-score (number of standard deviations above the mean of drug-naïve patients) produced by a particular *pol* genotype.

### Sequence Analysis of Real-Time PCR Target Regions and Control Real-Time PCR: Correction for Mismatch-Related Quantification Errors

To account for the possible effects of mismatches in the primer and probe binding regions on the efficiency of real-time PCR, prDNA extracted from patients' PBMC samples was used for sequence analysis of the target regions of prDNA and usRNA real-time PCR assays (these two assays are performed with the same set of seminested real-time PCR primers and probe). Regions corresponding to the *gag* gene of HIV-1 and containing the primer and probe binding sites were amplified in the nested PCR with the following primers: 5′GAG-1 (5′-CATGCGAGAGCGTCAGTATTAAGCGG-3′) and SK39 (5′-TTTGGTCCTTGTCTTATGTCCAGAATGC-3′) (gag-PCR I), and GAG-2I-SP6 (5′-CGATTTAGGTGACACTATAGGGGAAAAAATTCGGTTAIGGCC-3′) and GAGAE-3-T7 (5′-TAATACGACTCACTATAGGGACTATTTTATTTAATCCCAGGAT-3′) (gag-PCR II). The PCR products were sequenced directly with the primers of the gag-PCR II. Subsequently, the effects of mismatches in the primer and probe binding regions on the efficiency of real-time PCR were determined by performing the seminested real-time PCR with the patient-derived PCR amplicons that contained all the primer and probe target sites, as templates. The concentrations of the template amplicons were determined spectrophotometrically and equalized by dilution before real-time PCR. Patient-specific mismatch-related quantification errors (MRQE) were calculated as the differences between the log_10_-transformed output copy numbers of the individual mismatched templates and the log_10_-transformed median output copy number of the templates without mismatches. The presence of mismatches did not substantially influence the real-time PCR quantification, as the difference between the 10% and 90% percentiles of the MRQE values was 0.26 log_10_, and the total range, except for one outlier, was 0.34 log_10_ (Figure S1). There was no significant difference in MRQE values between the baseline samples and the corresponding under-therapy samples (*P* = 0.24, paired Wilcoxon signed rank test), and between the patient groups (*P* = 0.17, Mann-Whitney test).

### Statistical Analyses

For cellular and plasma HIV-1 load, statistical analyses were performed on log_10_-transformed values. All quantified amounts of prDNA and usRNA in the baseline and under-therapy PBMC samples were normalized to the individual MRQE values (see above), and statistical analyses were based on the normalized values. For the four PBMC samples in which prDNA and/or usRNA were undetectable, the prDNA and usRNA levels were left-censored at the corresponding detection limits, normalized to the MRQE values. For the comparisons of the baseline parameters between the patient groups, we used Mann-Whitney test for continuous variables and Fischer's exact test for discrete variables. For the comparisons of the levels of usRNA and prDNA and CD4^+^ count in patients on cART, under-therapy PBMC samples were divided in four groups based on sampling time from cART initiation: group I, 0.1–0.6 years; group II, 0.7–1.5 years; group III, 1.9–2.5 years; group IV, 2.9–6.0 years. Any of these groups did not contain more than one value from any patient. In three cases, patients were sampled twice in the period corresponding to the groupings (two cases in group IV and one in group I). In these cases, mean values per patient were used for the statistical analysis. The parameters were compared between baseline and time on cART-grouped PBMC samples by using paired Wilcoxon test and between failures and successes by using RM-ANOVA. Baseline values were excluded from the latter analysis. Longitudinal trends of the studied parameters under cART were estimated by fitting the linear mixed models, taking into account correlations of repeated measurements within the individual patients. Subsequently, linear mixed models were used to compare the virological parameters between the patient groups. The cutoff value of usRNA for the risk of VR was determined by the ROC curve analysis with therapy outcome as the classification variable and maximal value of usRNA under therapy as the diagnostic variable. Correlations of virological parameters and CD4^+^ count were assessed by using Spearman tests. All parameters, except for CD4^+^ count under therapy, were normally distributed (*P*>0.1, D'Agostino & Pearson omnibus normality test), and Pearson r^2^ values of pairwise correlations were calculated for these parameters. RM-ANOVA was performed by using XLSTAT (http://www.xlstat.com), logistic regression and ROC curve analysis by using MedCalc 10.4 (http://www.medcalc.be), and linear mixed model analysis by using SPSS 16.0 (http://www.spss.com/). All other statistical tests were performed by using GraphPad Prism 5.01 (http://www.graphpad.com). All statistical tests were two-sided. *P* values<0.05 were considered statistically significant.

## Supporting Information

Figure S1 Sequences of primer and probe target regions of seminested real-time PCR assays for usRNA and prDNA detection.
(0.07 MB PDF)

Table S1 HIV-1 load in PBMC and plasma and CD4^+^ counts.
(0.37 MB PDF)
