# Supplementary material for: Cellular Levels of HIV Unspliced RNA from Patients on Combination Antiretroviral Therapy with Undetectable Plasma Viremia Predict the Therapy Outcome
Source: PLoS One. 2009 Dec 31;4(12):e8490. doi: 10.1371/journal.pone.0008490 (PMC2795168; doi:10.1371/journal.pone.0008490)
Supplement: Figure S1 — Sequences of primer and probe target regions of seminested real-time PCR assays for usRNA and prDNA detection. (0.07 MB PDF) [file pone.0008490.s001.pdf]

	GAG1 (sense I, II)	GAG2 (antisense II)	GAG3 (probe)	SK431 (antisense I)	MRQE
REF	TCAGCCCAGAAGTAATACCCATGT	CACTGTGTTTAGCATGGTGTTT	ATTATCAGAAGGAGCCACCCCACAAGA	TGCTATGTCAGTTCCCCTTGGTTCTCT	
M10091	....	....	....	......A...C................	0.00
M10977	....	....	....	..........C................	0.02
M12735	....	....	....	..........C.............C..	-0.03
M12814	....	......R...............	....	..........C................	-0.01
M12852	..........R.............	......A..C........A...	........................G..	..........C................	-0.53
M12860	....	....	....	..........C................	0.00
M12865	.............G..........	...........R..........	....	..........C................	-0.10
M14693	....	....	....	..........C................	-0.02
M14741	.Y......................	....	....	..........C................	-0.03
M14758	....	....	....	..........C................	-0.12
M14769	.Y........R.............	...Y..................	.........R..............R..	..........C................	-0.18
M14785	.Y......................	....	....	......A...C................	-0.15
M17561	....	....	....	......A...C................	-0.07
M14453	.T..T...................	....	....	..........C................	0.00
M12794	.............R..........	...C..................	....	..........C................	-0.23
M14602	...AT........G..........	....	....	..........C................	-0.21
M14705	....	................A.....	.C.........................	..........C..........G.....	-0.05
M14732	.Y......................	......A...............	....	..........C................	-0.04
M11835	....	....	....	..........C................	-0.07
M12862	....T...................	....	....	..........C................	0.03
M14464	....	....	....	..........C................	0.02
M14639	.Y......................	....	........................G..	..........C................	-0.02
M14659	....	....	....	......A..GC................	0.04
M14760	....	...........A..........	....G......................	......A...C................	-0.13
M14858	.......R................	...........R..........	....	..........C................	-0.06
M14780	....	....	....	..........C................	0.11

Figure S1. Sequences of primer and probe target regions of seminested real-time PCR assays for usRNA and prDNA detection. Patient-derived sequences are compared with sequences of corresponding primers or probe (designated by “REF”). Patient numbers are given in the left column, and corresponding MRQE (mismatch-related quantification errors) are shown on the right. All primers and probe are in 5’-3’ orientation.
