# Supplementary material for: Cellular Levels of HIV Unspliced RNA from Patients on Combination Antiretroviral Therapy with Undetectable Plasma Viremia Predict the Therapy Outcome
Source: PLoS One. 2009 Dec 31;4(12):e8490. doi: 10.1371/journal.pone.0008490 (PMC2795168; doi:10.1371/journal.pone.0008490)
Supplement: Table S1 — HIV-1 load in PBMC and plasma and CD4+ counts. (0.37 MB PDF) [file pone.0008490.s002.pdf]

Table S1. HIV-1 load in PBMC and plasma and CD4⁺ counts

Patient group	Patient ID	Follow-up period, months	Study month[a]	Plasma RNA, $\log_{10}$ copies/ml	PrDNA, $\log_{10}$ copies/10^6 PBMC	UsRNA, $\log_{10}$ copies/µg total RNA	MsRNA, $\log_{10}$ copies/µg total RNA	$CD4^+$ T cell count, cells / mm^3 blood
F[b]	M10091	30.85	0	2.53	n/a[c]	n/a	n/a	140
			3.57	<1.70[d]	2.68	4.24	n/d[e]	270
			15.48	<1.70	2.66	3.85	n/d	320
			27.87	<1.70	1.77	2.98	n/d	400
F	M10977	25.97	0	5.34	n/a	n/a	n/a	100
			24.79	<1.70	3.02	3.82	n/d	250
F	M11835	18.33	0	3.60	2.91	3.82	n/d	280
			1.84	<1.70	3.11	3.45	n/d	580
			4.72	<1.70	3.00	3.25	n/d	470
			10.56	<1.70	3.66	3.26	n/d	680
F	M12735	41.84	0	4.00	4.20	4.01	2.63	280
			29.70	<1.70	2.79	3.40	n/d	730
			39.11	<1.70	3.03	4.17	n/d	470
F	M12794	20.69	0	4.97	4.60	4.71	3.08	140
			6.89	<1.70	4.36	4.56	2.29	360
F	M12814	47.64	0	3.88	3.02	4.62	2.85	150
			44.46	<1.70	2.26	3.02	n/d	530
F	M12860	28.85	0	4.59	3.64	5.53	3.12	120
			25.90	<1.70	2.48	4.29	2.84	230
F	M12865	14.69	0	5.61	3.45	5.16	3.20	170
			11.93	<1.70	3.43	3.67	n/d	360
F	M14453	18.26	0	3.41	3.91	4.40	2.69	300
			0.92	<1.70	3.14	3.48	n/d	280
F	M14464	30.92	0	4.35	3.99	4.53	3.22	270
			14.16	<1.70	3.43	3.64	n/d	550
			27.97	<1.70	3.50	3.10	n/d	390
F	M14639	37.41	0	4.91	4.13	4.45	2.81	250
			17.97	<1.70	3.69	3.16	n/d	420
			34.43	<1.70	3.55	3.53	n/d	640
F	M14659	12.95	0	4.76	4.41	5.01	3.51	280
			5.61	<1.70	3.93	3.44	n/d	500

			11.11	<1.70	3.64	3.16	1.96	510
F	M14741	7.41	0	4.84	4.39	5.18	3.89	480
			6.03	<1.70	3.59	3.44	n/d	380
F	M14858	15.31	0	4.36	3.68	4.13	2.89	270
			8.89	<1.70	3.67	3.72	2.49	480
F	M17561	41.51	0	5.15	3.81	4.08	n/d	60
			29.15	<1.70	3.00	3.84	1.82	750
			38.33	<1.70	3.04	4.02	n/d	850
S[f]	M12852	72.52	0	4.48	2.31	3.91	3.30	350
			5.51	<1.70	<1.82[g]	3.01	n/d	390
			43.80	<1.70	<1.63	<2.38	n/d	490
			72.52	<1.70	<1.91	<2.51	n/d	750
S	M12862	25.74	0	4.71	4.41	4.84	n/d	160
			3.44	<1.70	3.99	3.42	n/d	310
			13.57	<1.70	3.51	3.30	n/d	470
			25.74	<1.70	3.22	3.41	n/d	550
S	M14602	29.74	0	4.53	4.00	4.44	n/d	380
			3.15	<1.70	3.41	<2.21	n/d	490
			18.26	<1.70	3.23	3.19	n/d	1040
			29.74	<1.70	3.47	3.07	n/d	620
S	M14693	54.98	0	3.90	3.46	4.98	3.74	460
			14.23	<1.70	2.79	3.43	n/d	630
			54.98	<1.70	2.91	2.74	1.98	820
S	M14705	24.95	0	4.11	4.02	4.45	2.53	640
			3.61	<1.70	3.04	2.72	n/d	820
			13.74	<1.70	2.37	2.84	n/d	810
			24.95	<1.70	2.24	2.78	n/d	1190
S	M14732	15.84	0	4.48	4.22	4.23	3.06	480
			1.84	<1.70	3.39	2.91	n/d	610
			15.84	<1.70	3.72	3.04	n/d	730
S	M14758	47.70	0	5.67	3.64	5.99	3.01	370
			47.70	<1.70	2.81	3.68	n/d	170
S	M14760	23.28	0	4.30	3.75	4.14	2.84	210
			11.80	<1.70	3.27	3.51	n/d	420
			23.28	<1.70	3.41	3.45	2.15	420
S	M14769	57.38	0	3.85	3.28	4.84	2.67	190

			16.82	<1.70	2.63	3.61	n/d	320
			57.38	<1.70	2.38	4.20	2.39	700
S	M14780	26.26	0	4.50	4.13	4.50	2.92	250
			5.41	<1.70	3.76	3.45	n/d	350
			26.26	<1.70	3.61	2.71	n/d	380
S	M14785	59.02	0	4.30	3.04	5.29	4.09	650
			48.46	<1.70	2.52	2.84	n/d	1750
			59.02	<1.70	1.93	3.26	n/d	940

[a] Baseline (time of cART initiation) was taken as zero time point for every patient.
[b] F, future failures on cART.
[c] n/a, not applicable. No baseline PBMC samples were available for patients M10091 and M10977.
[d] 1,70 $\log_{10}$ copies/ml plasma corresponds to 50 copies/ml plasma (limit of detection of modern commercial assays).
[e] n/d, not detected.
[f] S, successfully treated patients.
[g] For the four PBMC samples in which prDNA and/or usRNA were undetectable, detection limits of the corresponding assays are shown.
